# Evaluation of the Diode laser (810nm,980nm) on dentin tubule diameter following internal bleaching

**DOI:** 10.4317/jced.52666

**Published:** 2016-07-01

**Authors:** Nazanin Kiomarsi, Soheil Salim, Pegah Sarraf, Mohammad Javad-Kharazifard, Nasim Chiniforush

**Affiliations:** 1 Assistant professor. Department of Operative Dentistry, Dental faculty, Tehran University of Medical Sciences, International campus, Tehran, Iran; 2 Doctor. Tehran University of Medical Sciences, International campus, Tehran, Iran; 3 Assistant professor. Department of Endodontics, Dental faculty, Tehran University of Medical Sciences, International campus, Tehran, Iran; 4 PhD candidate. Laser Research Center of Dentistry (LRCD), Dental Research Institute, Tehran University of Medical Sciences, Tehran, Iran

## Abstract

**Background:**

The aim of this study was to evaluate the effect of diode laser irradiation and bleaching materials on the dentinal tubule diameter after laser bleaching.

**Material and Methods:**

The dentin discs of 40 extracted third molar were used in this experiment. Each disc surface was divided into two halves by grooving. Half of samples were laser bleached at different wavelengths with two different concentrations of hydrogen peroxide. Other half of each disc with no laser bleaching remained as a negative control. Dentin discs were assigned randomly into four groups (n=10) with following hydrogen peroxide and diode laser wavelength specifications; Group 1 (30% - 810 nm), group 2 (30% - 980 nm), group 3 (46% - 810 nm) and group 4 (46% - 980 nm). All specimens were sent for scanning electron microscopic (SEM) analysis in order to measure tubular diameter in laser treated and control halves. Data were analyzed by ANOVA and Tukey test (*p*<0.05).

**Results:**

A significant reduction in dentin tubule diameter was observed in groups 1, 2 and 4. There was no significant difference between groups 1 and 2 and between groups 3 and 4 after bleaching.

**Conclusions:**

The SEM results showed that diode laser was able to reduce dentin tubule diameter and its effect on dentin was dependent on chemical action of bleaching material.

** Key words:**Laser, diode, dentin, tubule, diameter.

## Introduction

The origin of intrinsic discoloration of teeth is in the pulp chamber and arises from hemorrhage, necrosis, calcification and iatrogenic discoloration due to dental treatment (1). Internal bleaching is a conservative method for improving the shade of discolored non-vital teeth. In this method bleaching agents are placed in access cavity, in direct contact with dentin (2). The most commonly used internal bleaching technique is the walking bleach technique that bleaching agents; hydrogen peroxide and sodium perborate either alone or in combination are placed in pulp chamber. After some days, the bleaching result is evaluated and, if needed, bleaching agent is again inserted into the pulp chamber until favorable result is obtained (1). Owing to an increased demand for providing a real-time whitening result in a shorter period of time, different types of heat and light activation sources are used for catalyzing hydrogen peroxide decomposition and a faster whitening result. The process for activating whitening by heat and light is called thermocatalitic approach and photoxidation respectively (3). One of the newest light sources are lasers. The laser has been proven to be the most valuable energy source for in-office bleaching with simple and short application (4). A fundamental different between lasers and other light sources is that lasers emit a well-defined monochromatic light at a single wavelength in order to reduce possible side effects of other light sources such as overheating (5). The use of laser energy would be desirable from both safety and economic reasons because provide esthetic result with minimum exposure to hydrogen peroxide and in the minimum number of treatment sessions (6). Several diode laser systems with wavelength ranging from 790 nm to 980 nm are used for laser-assisted bleaching (7). The main advantage of the diode laser is its small size, portability and flexible optic fibers (8). There are few studies about the influence of diode laser and different wavelengths of this laser on tubular diameter changes of dentin (9). Many studies have shown that bleaching agents with different concentration caused chemical alteration in structures of human hard tissues and can alter the mineral contents (10,11). To the best of our knowledge, little data are available on the tubular diameter changes due to bleaching materials (12). Owing to the fact that the strength of the adhesive bond between the restorative material and dentin is affected by dentinal tubule diameter, as well as, the relative amount of intratubular and intertubular dentin (13) this study was designed to investigate any possible changes of dentin tubule diameter following internal laser bleaching. The purpose of this *in-vitro* investigation was to determine the effect of hydrogen peroxide (30% and 46%) and diode laser (810 nm and 980 nm) on dentinal tubule diameter by scanning electron microscopy (SEM). This would allow the investigation of site-specific morphological changes before and after bleaching techniques.

## Material and Methods

-Preparation of specimens

40 freshly extracted, decay-free human third molars were used in this experiment. This *in vitro* study was approved by Ethic committee of Tehran University of Medical sciences, International campus. The teeth were cleaned of any soft tissue covering the root surface. Teeth with any visible crack, fractures or any restorations and caries were excluded. Teeth were stored in distilled water at room temperature until laser application time. The root of each tooth was embedded in acrylic resin up to cementoenamel junction. Occlusal surfaces were cut at 4mm apical to the cusp tip with the slicing plan perpendicular to long axis of root (parallel to the occlusal surface) using a water-cooled diamond saw (precision wire diamond saw, Germany). A flat dentin surface on remaining tooth was exposed. Then each tooth sectioned to produce one dentin disc of 2 mm thick from each tooth, yielding a total of 40 discs. Each specimen was divided into two halves by grooving with bur. One half of each disc with no laser bleaching remained as a negative control. Specimens were randomly assigned into 4 groups (n=10).

-Bleaching procedure

Just before laser irradiation the smear layer was removed ultrasonically by a 17% EDTA solution for 2 min, followed by thorough rinsing with water to expose the dentinal tubules. Two bleaching materials selected for laser bleaching. The JW power bleaching gel by Heydent (Farafan diagnostics co, Tehran-Iran, under license of Heydent, Germany) which was mixed from a powder and a 30% hydrogen peroxide solution for the groups 1 and 2, the laserwhite 20 bleaching gel (Biolase, USA) which was a 46% hydrogen peroxide gel for the groups 3 and 4. Activation of bleaching gels occurred with a diode laser in two different wavelengths; at 810 nm (Cheese TM, Wuhan Gigaa Optronics Technology Co, LTD, China) for the groups 1, 3 and at 980 nm (Wiser, Dr. smile, Italy) for the groups 2, 4. A uniform layer of bleaching gel was attached on one half of dentin surface of each sample forming a layer about 2 mm thick and irradiation applied in the non-contact mode with an output power of 1.5 W, in continuous wave mode. Laser radiation was applied by a single practitioner for all samples and all irradiation factors in each group were done on the basis of the manufacturers’ recommendations. After carrying out laser bleaching protocols, discs were rinsed and stored in distilled water at 370Cfor 24 hours.

-Scanning electron microscopy

After bleaching, specimens were dehydrated before being prepared for the SEM. Then discs were fixed on aluminum stubs and sputter-coated with gold in a coater device (KYKY SBC-12). Specimens were examined at 500x and 2000x magnification with scanning electron microscope (KYKY EM3200) with working condition of 26KV. For each sample, tubular diameter calculations were done on both sides of each specimen with site selection based on having the same distance from the separating groove. The measurement of the smallest diameter across the tubule orifice minimized the error caused by tubules cut obliquely. Calibration was based upon the “scale-bar” (Philips) that was the software running on SEM. Afterwards, tubular diameter was calculated for each side of all specimens and these values were compared for determining tubular diameter change in each sample. Statistical analysis was performed using one-way analysis of variance (ANOVA), Tukey test and paired t-test.

## Results

**Figure 1:**
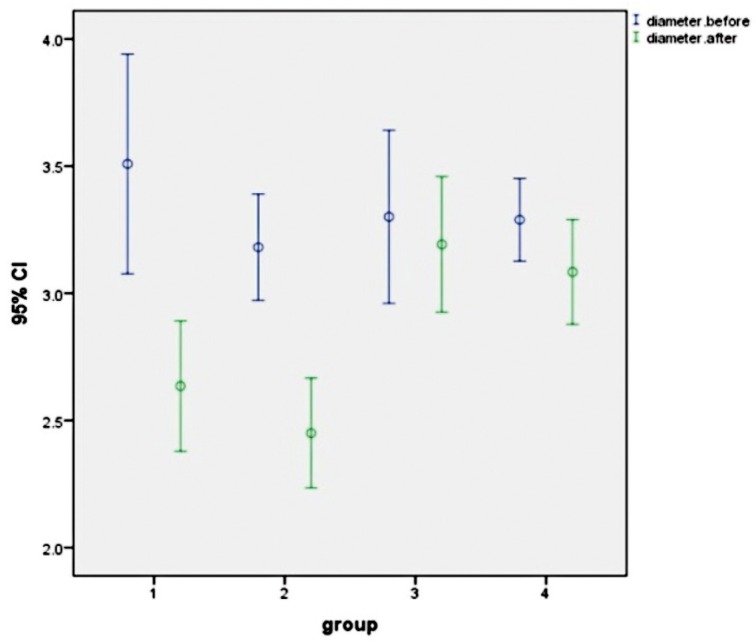
The comparison of mean ± Std. deviation of the dentinal tubule diameter of all groups before and after treatment.

The mean (and the Std. deviation) values of the dentinal tubule diameters for each group before and after laser bleaching are presented in  Table 1 and figure 1. Tubular diameter changes after bleaching were compared using repeated measure ANOVA while considering between subject factors. Afterwards, Tukey HSD multiple comparison test and a paired t-test were used for pair-wise comparisons and for understanding the changes in each group respectively. Analyses were done by SPSS 22 software. The level of confidence established at α=0.05. The tubular diameters before laser bleaching was not different between groups (*p*=0.391). The result of ANOVA showed a significant difference between groups after bleaching (*p*=0.000). Paired sample test revealed that reduction of tubular diameter after bleaching in all groups were significant with the exception of the third group (*p*=0.137). There was no significant difference in tubular diameter between groups 1 and 2 and between groups 3 and 4 after bleaching. SEM photos show the dentin surface changes after any laser bleaching protocols. The non-irradiated control areas presented a standard pattern of dentin treated by EDTA with open tubules and absence of smear layer. While in Irradiated areas partial obturation and in some areas narrowing and even a total occlusion of dentinal tubules were observed.

**Table 1:**
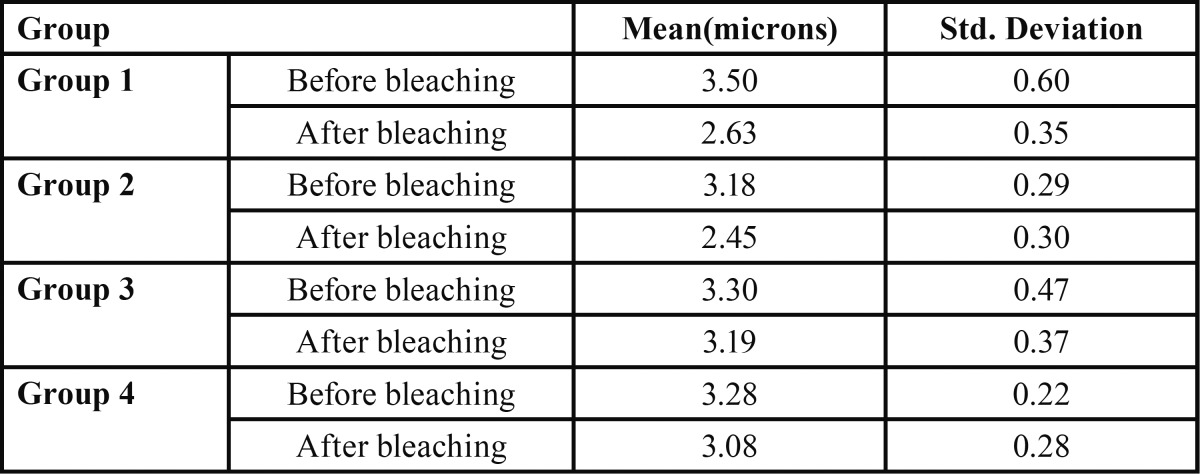
The mean ± Sd. deviation of the dentinal tubule diameter of all groups.

## Discussion

The composition of human dentin is about 70% mineral, 20% organic matrix and 10% water on a weight basis. The mineral content of dentin is composed primarily of hydroxyapatite crystallites. Dentinal tubules are small canals that extend the entire width of dentin and each dentinal tubule is lined with a layer of peritubular dentin, which is more mineralized than surrounding intertubular dentin (14,15).

Hydrogen peroxide as a whitening agent has ability to penetrate tooth structure. H2O2 is a strong oxidizing agent and it is also acidic that can affects dentin structure (15). Changes in organic matrix of dentin is mainly because of the oxidizing ability of hydrogen peroxide (through the formation of free radicals) whereas dentin demineralization and changes in mineral components are probably because of its acidity (15,16). During tooth bleaching, hydrogen peroxide by the action of free radicals (as a strong oxidizing agent) attacks the complex pigmented organic molecules responsible for tooth discoloration and converts them to simpler molecules which reflect less light (9). Alteration in human dentin regarding surface topography, hardness and mineral content have been observed after application of bleaching materials (11,12,15,17).

Near infrared (NIR) lasers such as diode lasers have a good penetration potential that allows the propagation of light through dentin. Diode lasers are poorly absorbed by dental hard substances (18). The wavelength of a laser determines its level of absorption. Diode lasers with wavelength of between 800 and 980 nm have limited interaction with water and hydroxyapatite but have high absorption peak for chromophores such as melanin, hemoglobin, and other pigmented proteins and carbonated hydroxyapatite (10,19). The FDA approved the use of diode lasers for bleaching procedures (8). The power output of these lasers ranges from 0.5 to 7 W and is delivered in two operating modes: continuous wave and pulsed mode (20).

The main effect of laser energy is the photo-thermal effect that converts light energy into heat (21). Furthermore it has photochemical effect that is based on photon absorption by specific photo-initiators present inside the bleaching agents which are adjusted to absorb the wavelength of the light source. These effects during laser bleaching help improving the potential of tooth whitening (22).

It is crucial to know the exact wavelength and energy of the laser irradiation. Ultra structural alteration in dentin irradiated with laser depends mainly on laser parameters (23). Laser-tissue interactions are strongly determined by the laser wavelength and applied power density (24). Different wavelength promote different tissue interaction and the dependency of the surface modification of tooth dentin on this parameter is not yet fully understood.

In the present study, laser activation of the bleaching agents was carried out by the diode laser with two different wavelengths (810 and 980 nm) and the chosen mode was continuous wave for easier scanning of whole dentin. One of the key factors in whitening efficacy from bleaching materials is the concentration of the peroxide. The bleaching agents tested in this study contained hydrogen peroxide in two different concentrations (30% and 46%).

Our results for dentinal tubule diameters before laser application are close to the means described in the literature (25-27). The results of this study showed that in all laser treated groups a decrease in tubular diameter was observed. This may be due to the melting effect of diode lasers as a result of thermal effects on surface dentin (28). Part of laser energy (especially 980 nm) is absorbed by mineral structures of dentin which provokes a sufficient increase in temperature that results in thermochemical ablation and melting of the dentin tissue (10). In the present study tubular diameter reduction by different wavelength of diode laser in groups with same bleaching material was not significantly different. Both wavelengths produced dentinal melting, narrowing and diameter reduction of the dentinal tubules.

The results of this investigation revealed that the effect of the diode laser on dentin is dependent on the chemical action of bleaching material that is in agreement with previous studies (19). Reduction in dentinal tubule diameter in groups 3 and 4 where 46% hydrogen peroxide was used as bleaching material was less than groups 1 and 2 in which 30 % hydrogen peroxide was applied. That can be attributed to the different hydrogen peroxide concentration and possible different acidity of bleaching materials. The acidity provided by the low PH of hydrogen peroxide solutions might have contributed for dentin demineralization and enlargement of dentinal tubules. Peritubular dentin is likely to affected predominantly by the PH of hydrogen peroxide (15). Apparently with higher concentration of hydrogen peroxide and resulting penetrating ability, the demineralization would be more (17). The decrease of dentinal tubule diameter yielded by diode laser could be of benefit by postoperative pain reduction in vital teeth but in endodontically treated teeth this effect is not in favor of bonding process (29).

## Conclusions

Under the limitations of the present study it can be concluded that 810 and 980 nm diode laser irradiation (1.5 W, continuous mode,) during laser bleaching produce dentinal melting and can lead to reduction of dentinal tubule diameter. This effect of the diode laser on dentin is dependent on concentration and acidity of bleaching material.

## References

[1] Zimmerli B, Jeger F, Lussi A (2010). Bleaching of nonvital teeth. A clinically relevant literature review. Schweiz Monatsschr Zahnmed.

[2] Amato M, Scaravilli MS, Farella M, Riccitiello F (2006). Bleaching teeth treated endodontically: long-term evaluation of a case series. J Endod.

[3] Hahn P, Schondelmaier N, Wolkewitz M, Altenburger MJ, Polydorou O (2013). Efficacy of tooth bleaching with and without light activation and its effect on the pulp temperature: an in vitro study. Odontology.

[4] Gurgan S, Cakir FY, Yazici E (2010). Different light-activated in-office bleaching systems: a clinical evaluation. Lasers Med Sci.

[5] Buchalla W, Attin T (2007). External bleaching therapy with activation by heat, light or laser-a systematic review. Dent Mater.

[6] Anaraki SN, Shahabi S, Chiniforush N, Nokhbatolfoghahaei H, Assadian H, Yousefi B (2015). Evaluation of the effects of conventional versus laser bleaching techniques on enamel microroughness. Lasers Med Sci.

[7] Wetter NU, Barroso M, Pelino JE (2004). Dental bleaching efficacy with diode laser and LED irradiation: an in vitro study. Lasers Surg Med.

[8] Coluzzi DJ (2004). Fundamentals of dental lasers: science and instruments. Dent Clin North Am.

[9] Gholami GA, Fekrazad R, Esmaiel-Nejad A, Kalhori KA (2011). An evaluation of the occluding effects of Er; Cr: YSGG, Nd: YAG, CO2 and diode lasers on dentinal tubules: a scanning electron microscope in vitro study. Photomed Laser Surg.

[10] Umana M, Heysselaer D, Tielemans M, Compere P, Zeinoun T, Nammour S (2013). Dentinal Tubules Sealing by Means of Diode Lasers (810 and 980 nm): A Preliminary In Vitro Study. Photomed Laser Surg.

[11] de Oliveira DP, Teixeira EC, Ferraz CC, Teixeira FB (2007). Effect of intracoronal bleaching agents on dentin microhardness. J Endod.

[12] Maleknejad F, Ameri H, Kianfar I (2012). Effect of intracoronal bleaching agents on ultrastructure and mineral content of dentin. J Conserv Dent.

[13] Marshall GW Jr, Marshall SJ, Kinney JH, Balooch M (1997). The dentin substrate: structure and properties related to bonding. J Dent.

[14] Perdigão J (2010). Dentin bonding-variables related to the clinical situation and the substrate treatment. Dent Mater.

[15] Chng HK, Ramli HN, Yap AU, Lim CT (2005). Effect of hydrogen peroxide on intertubular dentine. J Dent.

[16] Jiang T, Ma X, Wang Y, Zhu Z, Tong H, Hu J (2007). Effects of hydrogen peroxide on human dentin structure. J Dent Res.

[17] Carrasco LD, Fröner IC, Corona SA, Pécora JD (2003). Effect of internal bleaching agents on dentinal permeability of non-vital teeth: quantitative assessment. Dent Traumatol.

[18] Fried D, Glena RE, Featherstone JD, Seka W (1995). Nature of light scattering in dental enamel and dentin at visible and near-infrared wavelengths. Appl Opt.

[19] Faria MIA, Sousa-Neto MD, Souza-Gabriel AE, Alfredo E, Romeo U, Silva-Sousa YT (2013). Effects of 980-nm diode laser on the ultrastructure and fracture resistance of dentine. Lasers Med Sci.

[20] Coluzzi DJ (2000). An overview of laser wavelengths used in dentistry. Dent Clin North Am.

[21] Gontijo IT, Navarro RS, Ciamponi AL, Miyakawa W, Zezell DM (2008). Color and surface temperature variation during bleaching in human devitalized primary teeth: an in vitro study. J Dent Child (Chic).

[22] Luk K, Tam L, Hubert M (2004). Effect of light energy on peroxide tooth bleaching. J Am Dent Assoc.

[23] Heya M, Sano S, Takagi N, Fukami Y, Awazu K (2003). Wavelength and average power density dependency of the surface modification of root dentin using an MIR-FEL. Lasers Surg Med.

[24] Sulieman M (2005). An overview of the use of lasers in general dental practice: 1. Laser physics and tissue interactions. Dent Update.

[25] Schilke R, Lisson JA, Bauss O, Geurtsen W (2000). Comparison of the number and diameter of dentinal tubules in human and bovine dentine by scanning electron microscopic investigation. Arch Oral Biol.

[26] Lenzi TL, Guglielmi CdeA, Arana-Chavez VE, Raggio DP (2013). Tubule density and diameter in coronal dentin from primary and permanent human teeth. Microsc Microanal.

[27] Nakamichi I, Iwaku M, Fusayama T (1983). Bovine teeth as possible substitutes in the adhesion test. J Dent Res.

[28] Parker S (2007). Verifiable CPD paper: laser-tissue interaction. Br Dent J.

[29] Savadi Oskoee S, Alizadeh Oskoee P, Jafari Navimipour E, Ahmad Ajami A, Pournaghi Azar F, Rikhtegaran S (2013). Comparison of the Effect of Nd:YAG and Diode Lasers and Photodynamic Therapy on Microleakage of Class V Composite Resin Restorations. J Dent Res Dent Clin Dent Prospects.

